# Benefits for Dominant Red Deer Hinds under a Competitive Feeding System: Food Access Behavior, Diet and Nutrient Selection

**DOI:** 10.1371/journal.pone.0032780

**Published:** 2012-03-05

**Authors:** Francisco Ceacero, Andrés J. García, Tomás Landete-Castillejos, Jitka Bartošová, Ludek Bartoš, Laureano Gallego

**Affiliations:** 1 Departamento de Ciencia y Tecnología Agroforestal y Genética, ETSIA, Universidad de Castilla-La Mancha, Albacete, Spain; 2 Department of Ethology, Institute of Animal Science, Praha, Czech Republic; 3 Sección de Recursos Cinegéticos, IDR, Universidad de Castilla-La Mancha, Albacete, Spain; 4 Animal Science Techniques Applied to Wildlife Management Research Group, IREC Sec. Albacete, Campus UCLM, Albacete, Spain; Cajal Institute, Consejo Superior de Investigaciones Científicas, Spain

## Abstract

Social dominance is widely known to facilitate access to food resources in many animal species such as deer. However, research has paid little attention to dominance in *ad libitum* access to food because it was thought not to result in any benefit for dominant individuals. In this study we assessed if, even under *ad libitum* conditions, social rank may allow dominant hinds to consume the preferred components of food. Forty-four red deer hinds (*Cervus elaphus*) were allowed to consume *ad libitum* meal consisting of pellets of sunflower, lucerne and orange, and seeds of cereals, corn, cotton, and carob tree. The meal was placed only in one feeder, which reduced accessibility to a few individuals simultaneously. During seven days, feeding behavior (order of access, time to first feeding bout, total time spent feeding, and time per feeding bout) were assessed during the first hour. The relative abundance of each meal component was assessed at times 0, 1 and 5 h, as well as its nutritional composition. Social rank was positively related to the amount of time spent feeding during the 1^st^ h (*P* = 0.048). Selection indices were positively correlated with energy (*P* = 0.018 during the 1^st^ h and *P* = 0.047 from 1^st^ to 5^th^) and fat (only during the 1^st^ h; *P* = 0.036), but also negatively with certain minerals. Thus, dominant hinds could select high energy meal components for longer time under an *ad libitum* but restricted food access setting. Selection indices showed a higher selectivity when food availability was higher (1^st^ hour respect to 1^st^ to 5^th^). Finally, high and low ranking hinds had longer time per feeding bout than mid ones (*P* = 0.011), suggesting complex behavioral feeding tactics of low ranking social ungulates.

## Introduction

Social dominance is widely known to facilitate access to food resources in many animal species, especially during food-shortage periods [1], [2]. Thus, a great interest has focused on the importance of social rank to obtain larger or better food resources under natural and captive conditions. Differential access to food related to social rank has been reported in several ungulates, including red deer (*Cervus elaphus*) stags [1] and hinds [3], [4]. However, other studies failed to find this relationship between social rank and food access when food was in large quantity and evenly distributed [5], [6]. Thus, dominance is supposed not to result in any benefit for dominant individuals under *ad libitum* access to food.

Preference for those meal components with highest nutritive quality has been also repeatedly shown for cervids [7], [8] under different experimental settings. Thus, provision of a ‘Total Mixed Ration’ is a common practice since it is supposed to decrease sorting of individual ration components and to promote a more balanced intake of nutrients among the herd [9]–[11]. However, under restricted access to feeders, dominant hinds would still benefit by selecting the most nutritive ration through a preferential access even if food is offered *ad libitum*, although this has not been previously proved.

In this study, we designed an experimental setting with *ad libitum* but greatly competitive feeding conditions. This way, we tested simultaneously: a) if social rank allows dominant hinds a better access to feeders; and b) if hinds with preferential access select for certain meal components of the Total Mixed Ration. Finally, nutritional properties of every meal component used were examined in order to find which nutrients drive the observed meal selection.

## Materials and Methods

### Ethics Statement

The experiment was designed according to European and Spanish laws and current guidelines for ethical use of animals in research [12]. All experimental procedures were conducted under the approval of the University of Castilla-La Mancha Animal Ethics Committee. Fresh water was always available through automatic dispensers. Wood shelters and straw litters were available to ensure animal welfare.

### Animals and Housing

This study was carried out at the experimental enclosures for deer research of the University of Castilla-La Mancha in Albacete (Spain) in March 2009. These facilities were established in 1994, and most of the animals currently kept (including all the hinds used in this experiment) are born in captivity. Study subjects were a herd of 44 adult Iberian red deer hinds between 5 and 13 years of age kept captive in a 15,000 m^2^ paddock with bare soil. All hinds were in late pregnancy, which is one of the most demanding stages of reproduction for female mammals [13] and requirements for all nutrients increase [14]. Thus, competition for food and nutrients in our experiment should be greater than in other stages. Previous studies in other groups have shown that 90% of red deer calve within one month [15], and we obtained similar results in our farm. This means that the hinds studied could differ in pregnancy stage only a few weeks. Nevertheless, the experiment was done during the second third of gestation. Many studies show that the greatest energetic needs of gestation and weight gains of the fetus occur during last third of pregnancy [13], [16]. Thus, requirements should be high, but differences in requirements among hinds should be unlikely to greatly influence the results, as it would have been the case in the last third of gestation.

Red deer hinds establish stable social hierarchies mainly based on age [17]. Thus, a wide age ranged (5 to 13 years) hinds group was selected to be studied in order to get a group with a social hierarchy as stable as possible. Nevertheless, younger hinds were avoided in the studied group since these hinds may have greater requirements (maintenance, pregnancy, plus extra requirements due to late growing; see [18] for an example of increased mineral requirements in young red deer hinds).

### ‘Restricted Food Access’ Experiment

During seven experimental days the herd was fed *ad libitum* with a mixture consisting of pellets of sunflower, lucerne and orange, and seeds of cereals, corn, cotton, and carob tree. Corn, garlic and oat straw were also continuously available as meal complement. Routinely, food is supplied once a day in several feeders. Diet was the same during the previous 3 months, so animals were just familiar with the nutritional value of the different meal components (see [19]).

Nutritive parameters (ash, starch, sugar, crude protein, fat, fibre, and energy) of every component in the diet were analyzed by an ENAC approved laboratory (Alkemi S.A., Coslada, Madrid). Samples of every component were also collected, dried and ground, and 0.6 g were used to assess mineral content. The powder obtained was dissolved with 3 mL of 63% HNO_3_, 1.5 mL of 37% HCl, and 5.5 mL of deionized water. A second wet digestion was carried out in the microwave oven (CEM MDS-2000, Barcelona, Spain) under the conditions of 345 kPa for 30 min. After cooling, the digestion solutions were transferred to volumetric flasks and 15 mL of deionized water was added for analysis. Samples were then examined with an atomic absorption spectrophotometer (Perkin-Elmer Plasma 400, Boston, MA, USA). Each datum was the mean of three measures recorded at 0.3 s intervals. Ca, K, Mg, Na, P, Si, B, Co, Cu, Fe, Mn, S, Se, Sr, and Zn were assessed for every component (see Table 1 for overall nutritional characteristics of diet, and Table 2 for percentage of every component in the initial whole meal).

**Table 1:** Nutritive and mineral mean (±SE) contents of offered wholemeal feed, and food remaining after red deer hinds fed for 1 and 5 hours.

	Start	1 h		5 h	
**Energy (KJul/Kg)**	8 714	7 219±300	t_1,6_ = 5.0 **	5 270±584	t_1,6_ = 3.0 *
**Crude Protein (%)**	11.2	12.0±0.5		10.5±1.8	
**Starch (%)**	27.0	17.2±2.4	t_1,6_ = 4.1 *	7.7±2.7	t_1,6_ = 3.2 *
**Fat (%)**	3.0	2.5±0.1	t_1,6_ = 5.6 **	1.9±0.2	t_1,6_ = 3.4 *
**Neutral Detergent Fibre (%)**	37.5	34.7±0.6	t_1,6_ = 4.3 **	27.9±3.1	t_1,6_ = 2.6 *
**Acid Detergent Fibre (%)**	13.8	16.8±0.9	t_1,6_ = −3.3 *	18.0±1.0	
**Sugar (%)**	6.4	7.7±0.4	t_1,6_ = −3.0 *	8.6±0.6	
**Ash (%)**	4.1	5.7±0.4	t_1,6_ = −4.4 **	6.8±0.4	t_1,6_ = −2.8 *
**Ca (%)**	0.801	1.680±0.177	t_1,6_ = −5.0 **	3.020±0.413	t_1,6_ = −4.0 *
**K (%)**	0.652	0.779±0.037	t_1,6_ = −3.4 *	0.832±0.052	
**Mg (%)**	0.132	0.152±0.007	t_1,6_ = −2.8 *	0.153±0.017	
**Na (%)**	0.039	0.058±0.006	t_1,6_ = −3.3 *	0.066±0.010	
**P (%)**	0.249	0.246±0.004		0.213±0.026	
**Si (%)**	0.148	0.133±0.0016	t_1,6_ = 2.5†	0.093±0.024	t_1,6_ = 2.1†
**S (%)**	1.729	1.999±0.103	t_1,6_ = −2.6 *	1.953±0.260	
**B (ppm)**	11.9	17.9±1.6	t_1,6_ = −3.8 *	23.1±1.8	t_1,6_ = −2.4†
**Co (ppm)**	0.80	0.88±0.02	t_1,6_ = −3.1 *	0.92±0.03	
**Cu (ppm)**	6.7	8.3±0.6	t_1,6_ = −2.7 *	8.4±1.2	
**Fe (ppm)**	204.0	279.6±25.3	t_1,6_ = −3.0 *	284.6±56.0	
**Mn (ppm)**	22.3	23.1±0.3	t_1,6_ = −2.3†	21.6±2.0	
**Se (ppm)**	7.1	7.3±0.0	t_1,6_ = −9.2 ***	7.5±0.1	t_1,6_ = −3.4 **
**Sr (ppm)**	29.9	44.1±3.1	t_1,6_ = −4.6 **	58.7±3.7	t_1,6_ = −2.9 **
**Zn (ppm)**	26.4	28.4±0.3	t_1,6_ = −2.3†	27.0±2.8	

The experiment was repeated for 7 days, and paired t-tests show differences between initial and 1^st^ hour food remaining, and between 1^st^ to 5^th^.

†, *, **, and *** respectively indicate significant differences at 0.1, 0.05, 0.01, and 0.001 level.

Only significant values in paired t-tests are shown.

**Table 2:** Meal components (mean ±SE) and selection indices during the 1^st^ feeding hour.

A)	Mixture at start (%)	1^st^ h mixture(%; *n* = 7)	Resourceuse 0 to 1^st^ h (%; *n* = 7)	Selection ratio (*ŵ* *^0–1^*)	Standarized ratio (*B^0–1^*)	κ^2^
**Sunflower Pellet**	14.7	26.2±3.8	10.2±4.1	0.700	0.107	10.324^ns^
**Corn**	9.8	5.1±0.4	11.5±0.5	1.175	0.180	1.433^ns^
**Orange Pellet**	12.2	31.4±3.3	4.9±3.7	0.406	0.062	**23.565 ** **
**Carob-Tree Seed**	8.1	8.1±0.8	8.1±0.8	0.998	0.153	0.291^ns^
**Cereal Grain**	53.0	26.2±6.8	63.1±7.0	1.189	0.182	**12.036** †
**Cottonseed**	0.71	0.32±0.04	0.86±0.05	1.201	0.184	0.144^ns^
**Lucerne Pellet**	1.5	2.6±1.3	1.3±0.8	0.870	0.133	1.651^ns^

A high *ŵ* (greater than 1) or *B* (greater than 1/7 = 1.43) indicates food items selected positively. A low *ŵ* (lower than 1) or *B* (lower than 1.43) indicates food items selected negatively.

† and ** respectively indicate if selection was significant at 0.1 and 0.01 level.

**Figure 1:**
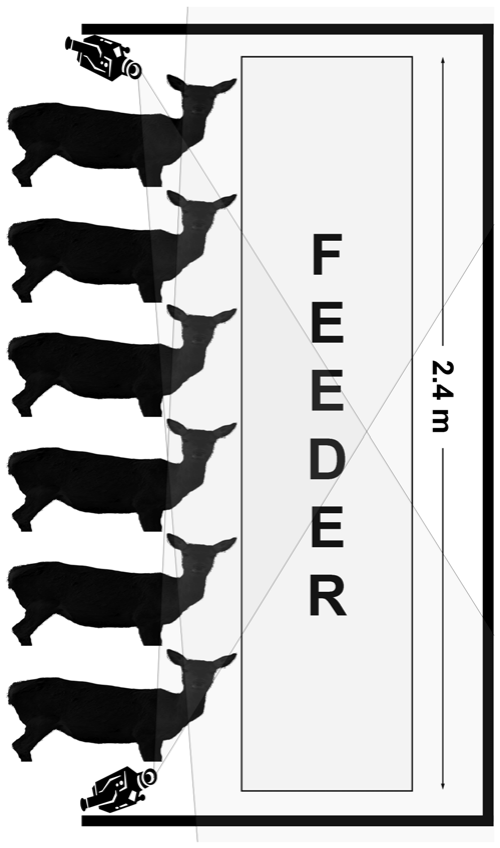
Experimental design for the ‘Restricted food access’ experiment. Only 6 hinds at most are allowed to feed at once. Location of video cameras and their visual field is shown.

During seven consecutive experimental days (9^th^ to 14^th^ March 2009) food was supplied only in one feeder with reduced accessibility (thus, 6 hinds at most could feed at once). Feeding behavior was recorded by video cameras from the time the meal was offered and for one hour out of the five that each session lasted (Figure 1). Every hind was marked with numbered collars (655×59×12 mm), so they could be individually identified (animals are routinely collared in the first weeks of life). Time spent feeding during the first hour (*FT*, in seconds), time to first feeding bout (*FFB*, in seconds; considering a feeding bout when a hind was successfully observed feeding), and order of access (*AO*, 1^st^ to 44^th^) was recorded for every hind and every experimental day. Total number of feeding bouts (*TFB*, which ranged from 1 to 31), and mean time per feeding bout (*T/n*, in seconds) was also assessed for every hind and every experimental day as a measure of the degree of relaxation/stress that every hind was enjoying/suffering when feeding under this competitive experimental setting. Even if the experiment was repeated during 7 consecutive days, only mean values for every hind and every variable was used in further analyses to avoid pseudoreplication. *FFB* and *T/n* were log-transformed to approach normality.

During the seven experimental days, the amount of food remaining after 1 and 5 hours was weighed with a Gram Precision AK Eagle 30 (±5 g) portable scale (Madrid, Spain). Initial relative amounts of every meal component were assessed by measuring the dry matter weight of every one in ten subsamples of the mixture (*c.* 100 g). The percentage remaining for every component after 1 (Table 2) and 5 hours (Table 3) was also assessed every experimental day as the mean value for 5 subsamples (*c.* 100 g), in order to examine the preferences for every component and the nutritive value of food fed during early feeding (first hour) and thereafter (first to fifth). Food was *ad libitum* since certain amount of food was already available at the start of every new trial (*i.e.*, 24 hours after the previous one).

**Table 3:** Meal components (mean ±SE) and selection indices from 1^st^ to 5^th^ hours.

B)	1^st^ h mixture(%; *n* = 7)	5^th^ h mixture(%; *n* = 7)	Resourceuse 1^st^ to 5^th^ h (%; *n* = 7)	Selection ratio (*ŵ* *^1–5^*)	Standarized ratio (*B^1–5^*)	κ^2^
**Sunflower Pellet**	26.2±3.8	25.5±6.8	24.7±13.9	0.958	0.126	2.331^ns^
**Corn**	5.1±0.4	1.5±1.0	6.4±2.1	1.226	0.161	0.653^ns^
**Orange Pellet**	31.4±3.3	60.0±10.8	23.0±12.1	0.755	0.099	5.010^ns^
**Carob-Tree Seed**	8.1±0.8	3.2±1.8	9.8±3.3	1.191	0.157	0.835^ns^
**Cereal Grain**	26.2±6.8	6.6±5.6	33.0±24.6	1.248	0.164	3.365^ns^
**Cottonseed**	0.32±0.04	0.12±0.07	0.39±0.13	1.191	0.157	0.034^ns^
**Lucerne Pellet**	2.6±1.3	3.1±1.3	2.8±3.5	1.028	0.135	0.264^ns^

A high *ŵ* (greater than 1) or *B* (greater than 1/7 = 1.43) indicates food items selected positively. A low *ŵ* (lower than 1) or *B* (lower than 1.43) indicates food items selected negatively. Any food item was significantly selected.

### Social Rank

Interactions to establish social rank were monitored during the previous week to the ‘restricted food access’ experiment, from March 2^nd^ to 7^th^ 2009. Although social hierarchy is considered to be stable in red deer hinds ([17]; but also in other wild ungulates [20]), the observation period was not extended for longer to avoid any small variation in the hierarchy during the experimental period. A total of 14 observation hours were carried out in 2 h periods during those moments with higher social activity [17]. All interactions were registered avoiding interferences in the behavior of the animals, according to the focal group sampling method [21]. The observer stayed hidden from the animals outside the enclosure, with optimal observation conditions. Following [22], agonistic interactions were considered as occasions when one hind physically attacked another one, or made a ritualized gesture associated with attacks that led to the other animal moving away. Threats included one or more of the following behaviors: butting with the forehead against the other's body; biting (usually directed towards the back or ears); kicking with the forelegs and chasing; all of which varied in intensity, sometimes being reduced to a mere intention movement in which no actual contact was made [23]. No serious injuries were observed during agonistic encounters.

Dominance rank for each individual was calculated as a linear hierarchy by winner–loser outcome of interactions on Matman 1.1.4 matrix manipulation and analysis program (Noldus, Wageningen, The Netherlands) as explained by [24]. This method was chosen respect to other proposed ones because it can be applied to a small sample size, as we had, with significant results. To determine the statistical significance of the linearity (*h*′) of the dominance hierarchy, a sampling process using 10 000 randomizations was performed [25]. Dominance hierarchy was reorganized by a two-step iterative procedure (1 000 sequential trials) to order individuals by first minimizing the number of inconsistencies, and thereafter the strength of the inconsistencies. Linear rank was transformed according to the formula 1–(*rank*/*n*) [*n* = 44]. Therefore, social ranks varied from 0 to 1. Finally, social rank value was transformed into arcsine of the square root to fit a normal distribution.

### Statistical Analyses

One-way Pearson correlations showed the effect of social rank on the behavioral indices of food access (*FFB*, *AO*, *TFB*, *FT*, and *T/n*). One-way procedure was selected since the direction of all the correlation was known in advance. This procedure showed if dominant hinds were actually getting some benefit in food access and resource selection. Thereafter, Curve Estimation Regression Models were performed to identify other non linear relationships between social rank and food access indices. This SPSS procedure test polynomial, logarithmic and other relationships at once.

To determine diet selection, a resource selection ratio (*ŵ*) was calculated for every meal component as *ŵ* *_i_ = o_i_/π_I_*, being *o_i_* the proportion of used resource *i*, and *π_i_* the proportion of available resource *i* (*sensu* [26]). Values *o_i_* and *π_i_* were the mean for the seven experimental days. This method was selected because it allows determining the significance of the observed resource selection (*ŵ*) by χ^2^ test [26]. In other commonly used indices (like Ivlev's [27]) the researcher subjectively decides which components are being selected, which may lead to errors (note in our results that some non significant components have a higher *ŵ* value than other significant ones). Selection ratio was assessed for both studied periods (during the start of the trial to the end of 1^st^ feeding hour, and during 1^st^ to 5^th^ hour).

To determine nutrient selection, one-tail Spearman's Rho correlations showed which nutrients were related to the selection ratios observed for every meal component in both studied periods (*ŵ* *_i_^0–1 h^* and *ŵ* *_i_^1–5 h^*). This procedure showed which nutrients were significantly related to a higher or lower component selection in every experimental period. For a better understanding of the relative importance of each nutrient in the observed selection of meal compounds, and to allow a comparison between the two studied periods, standardized selection ratios (*B_i_^0–1^, B_i_^1–5^*) were also calculated according to the formula *B_i_ = ŵ* *_i_/(Σŵ* *)*, so that they add to 1 [23]. Finally, paired t-tests showed differences between meal remaining after one hour respect to initially offered meal, and after five hours respect to meal remaining after the first one. This way, nutrient selection in the whole diet was assessed for every experimental period.

Statistical analyses were performed using SPSS statistical package (SPSS version 17.0, SPSS Inc., Chicago, IL). Since sample size is slightly low for certain analyses (e.g. 7 experimental days or 7 meal components), marginal significance (*P*<0.1) is also indicated so the reader will be able to reach own conclusions about these results.

## Results

### Social Rank and Food Access

**Figure 2:**
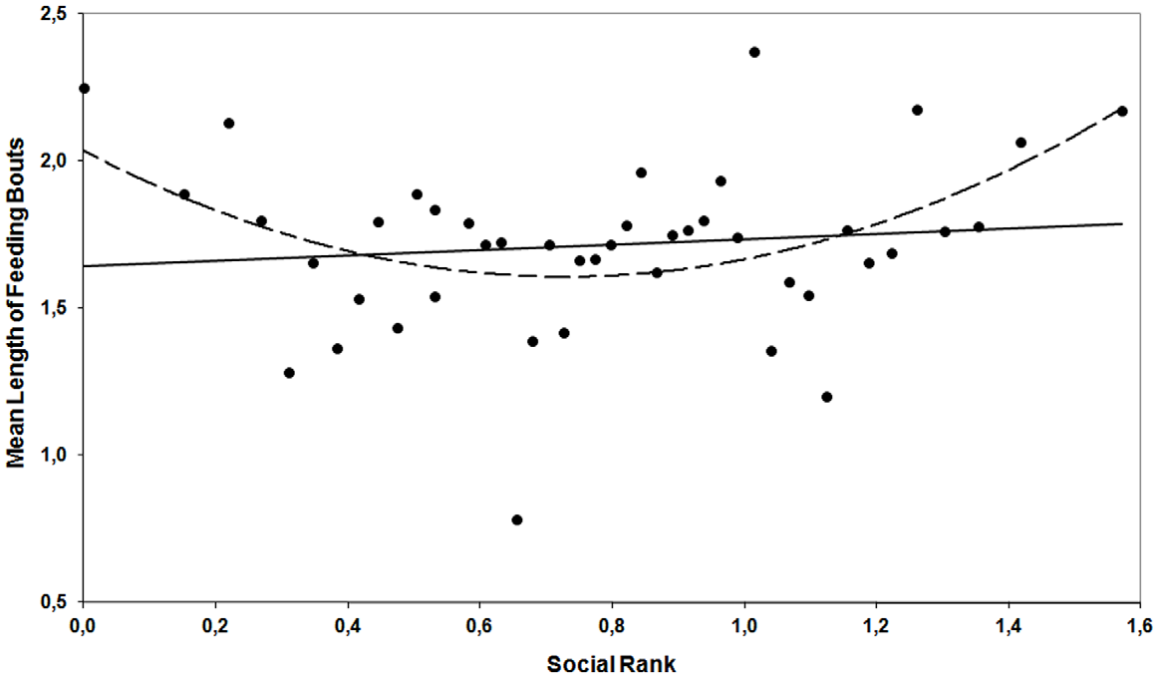
Plotting of *T/n* (mean time per feeding bout, log- transformed to achieve normality) *vs.* normalized social rank (*ArcsenSqrt* of hierarchical social rank; Côté 2000), with linear (solid line; *R* = 0.11; *P* = 0.230) and quadratic (dashed line; *R* = 0.44, *P* = 0.011) adjusting.

Females showed a significant linear hierarchy (*h′* = 0.107; *P* = 0.037) correlated with age (Pearson's correlation: *r* = 0.362, *n* = 46, *P* = 0.017). Behavioral indices of food access (*FFB*, *AO*, and *TFB*) correlated with each other (Table 4). *T/n*, which was a measure of the disturbance degree that animals were suffering, only correlated with *FT* (*i.e.*, total feeding time was longer for animals with longer feeding bouts). Social rank correlated positively with the total time that every animal spent feeding during the first hour (*FT*). However, social rank did not ensure earlier access to food (in time or order: absence of correlation of social rank with *FFB* and *AO*). Curve Estimation Regression Models also showed a quadratic relationship between social rank and *T/n* (Figure 2; *R* = 0.444; *F* = 5.024; *P* = 0.011; Coefficients: *ArcsenSqrtRank P* = 0.009, *ArcsenSqrtRank^2^ P* = 0.004; *i.e.*, both high and low ranking animals had longer feeding bouts than mid-ranked).

**Table 4:** One-way Pearson correlations among social rank and food access behavioral indices (*T/n* = mean time per feeding bout; *FFB* = time to first feeding bout; *AO* = order of access to the feeder; *TFB* = total number of feeding bouts; *FT* = time spent feeding during the first hour; *N* = 44).

	*Rank*	*T/n*	*FFB*	*AO*	*TFB*
***T/n***	0.114				
***FFB***	−0.027	0.074			
***AO***	−0.124	−0.304	**0.794 ** ***		
***TFB***	0.190	0.044	**−0.608 ** ***	**−0.720 ** ***	
***FT***	**0.254 ** *	**0.643 ** ***	**−0.297 ** *	**−0.550 ** ***	**0.552 ** ***

*, and *** indicate significance at 0.05, and 0.001 level.

### Food Selection

During the first feeding hour, only cereal grain was marginally positively selected (*P*<0.1), while orange pellets were negatively selected (*P*<0.01; Table 2). No meal component was significantly selected or avoided during the following period (1^st^ to 5^th^ hours; Table 3). Nevertheless, Spearman's Rho correlations between selection ratios and nutritive values of every meal component were found in both studied period (*ŵ* *_i_^0–1 h^* correlated with energy: *R* = 0.79, *P* = 0.018; fat: *R* = 0.71, *P* = 0.036; Ca *R* = −0.82, *P* = 0.012; and Sr *R* = −0.75, *P* = 0.026; *ŵ* *_i_^1–5 h^* correlated with energy: *R* = 0.68, *P* = 0.047; Ca: *R* = −0.89, *P* = 0.003; Na: *R* = −0.71, *P* = 0.036; B: *R* = −0.86, *P* = 0.007; Fe: *R* = −0.68, *P* = 0.047; and Sr: *R* = −0.68, *P* = 0.047). Selection ratios were highly homogeneous during the first period during the 7 experimental days (*ŵ* *_i_^0–1 h^*), especially for grains and seeds (CV^0–1 h^ = 4.2 for corn; 5.8 for cottonseed; 9.5 for carob-tree seeds; 11.1 for cereal grains) but lower for silages (CV^0–1 h^ = 39.8 for sunflower; 59.5 for lucerne; 74.6 for orange). Greater heterogeneity but similar pattern was observed during the second period (*ŵ* *_i_^1–5 h^*): CV^1–5 h^ = 21.9 for carob-tree seeds; 22.0 for cereal grains; 23.0 for corn; 23.8 for cottonseed; 44.9 for sunflower; 45.3 for lucerne; 69.0 for orange.

**Figure 3:**
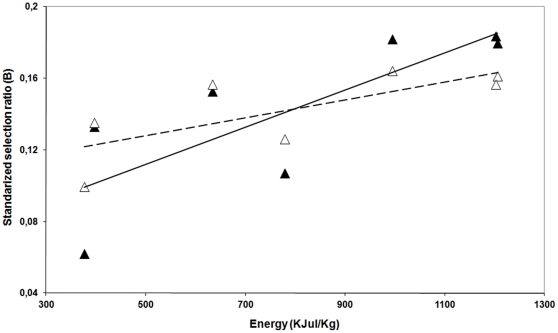
Influence of the energy on the observed standarized selection ratios during first feeding hour (*B_i_^0–1^*; black triangles and solid line; *R* = 0.79) and first to fifth (*B_i_^1–5^*; hollow triangles and dashed line; *R* = 0.68). Note the different slopes, which indicate a lower selectivity in the second period when the amount of food available was lower.

Standardized selection ratio increased in the second studied period (*B_i_^1–5 h^* respect to *B_i_^0–1 h^*) for those meal components with increased availability (sunflower, orange and lucerne silage), decreased for those with reduced availability (cottonseed, corn and cereal grains) and stayed stable when availability was similar (carob-tree seed; Tables 2 and 3). Thus, when comparing both periods selectivity for high-energy meal components was higher in the 1^st^ hour respect 1^st^ to 5^th^ (note change in slope for overall energy selectivity in Figure 3).

Finally, the observed diet selection patterns produced several significant and some marginally significant variations in the nutritive and mineral values of the wholemeal available at every stage (Table 1). Paired t-tests showed that food remaining after one hour had significantly less starch, fat, neutral detergent fibre and energy, and greater amount of most minerals, ash, sugar, crude protein and acid detergent fibre. Remaining food after five hours had significantly less starch, fat, neutral detergent fibre and energy, and more ash, Ca, Se, and Sr than remaining meal after one hour. Thus, those hinds feeding longer during the first period (first hour) actually selected for higher contents of energy, fat and starch than late feeders.

## Discussion

### Social Rank and Food Access

Differential access to food related to social rank has been reported in several ungulates, including red deer (*Cervus elaphus*) stags [1] and hinds [3]–[4], muskox (*Ovibos moschatus* [28]), reindeer (*Rangifer tarandus* [29]), chamois (*Rupricapra rupricapra* [30]), American bison (*Bison bison* [31]), Rocky Mountain goat (*Oreamnos americanus* [32]; [2] for salt blocks access), African buffalo (*Syncerus caffer* [33]), bighorn sheep (*Ovis canadiensis* [34]), dama gazelle (*Nanger dama* [35]), Cuvier's gazelle (*Gazella cuvieri* [35]), pronghorn (*Antilocapra Americana* [36]), Barbary sheep (*Ammotragus lervia* [37]); but also in farm ruminants as goats [38] and dairy cows [39].

In our experiment, social rank showed a linear correlation with the total time that hinds spent feeding during the first hour (similar results obtained by [40] in feeders for cows, and by [41] in supplementary feeding sites for red deer stags). Thus, dominant hinds may benefit for a longer time selecting meal components with greater energy content, which is consistent with previous studies in red deer and other captive or free-ranging ungulates [29], [30]. However, our results assess for the first time that social rank influences food access even under *ad libitum* feeding regimes of a balanced total mixed ration when feeding system force animals to compete. This ability of red deer hinds to compete for food resources may explain indirect influences (through body weight and condition) found for social rank over reproductive success (see [42] in a population subjected to winter food restriction; and [43] for similar effects in other ungulates), or offspring survival [44]. This indirect effect was also previously found for milk production under a lower level of competition for food [45], and thus, this effect is expected to be much higher under a more competitive feeding system.

Nevertheless, dominant hinds did not show an early access to food neither in time or order. Similar results were previously reported. Dubuc and Chapais [46] showed that arrival order did not correlate with dominance rank in *Macaca fascicularis* because subordinate animals used what these authors called ‘early arrival tactic’. This may be also happening in our experiment since *FFB* and *AO* both showed a non significant but negative correlation with social rank. In fact, although rank did not correlate with *T/n*, *FFB*, *AO* or *TFB*, dominant hinds spent longer time feeding, and those spending longer time feeding also showed an earlier access to the feeder and had a greater number of feeding bouts and a longer time per feeding bout.

Both low and high ranking hinds enjoyed a longer time per feeding bout than mid-ranked ones. Although this is a counterintuitive result, it is not a new pattern. Dennehy [36] observed that high and low rank *A. americana* females selected better quality diets under free ranging conditions. Veiberg et al. [47] also showed that feeding time in red deer hinds was only correlated with ranking in high but not in low and medium ranking animals. Dennehy [36] discussed that low rates of agonistic interaction or low vigilance times by low ranking hinds could explain this result. Vigilance has no sense in our study but agonistic interactions do. Aggressions increase when increasing the number of animals per feeder, and consistency of this encounter decrease [48]. Thus, the probability that a submissive displace a dominant hind one from the feeder increase, since individual recognition is more difficult because of the low visibility (head down and surrounded by other hinds). Thus, dominant individuals often react as submissive: [2] showed that *O. americanus* competing for mineral licks first react as subordinate because they cannot recognize the identity of the aggressor, attacking subsequently if the aggressor was subordinate and regaining the preferential position. Thus, the most submissive hinds may be less reactive to aggressions and stay feeding as long as possible (enjoying a higher *T/n*) if they have a stronger drive to consume food as a result of their lower body condition. Friendship among animals (studied through allogrooming behavior) was shown to be also correlated with partners' preferences in feeders [49]. Finally, another possibility that deserves to be further studied is that dominant hinds displace mid ranking animals more frequently than low ranking ones because the risk of losing social status increase if mid ranking hinds take advantage or preferential access to resources. Thus, further studies on behavioral feeding tactics both in captive and free ranging social ungulates are needed to understand these strategies [36], [47].

### Food Selection

Feeding under a competitive experimental setting, red deer hinds showed a marginally positive selection of cereal grains and negative selection of orange pellets during the 1^st^ feeding hour. Subsequently (from 1^st^ to 5^th^), food selected was not significantly different than food offered. However, these results should be considered with caution, since significance is expected to increase when increasing the amount of food offered and the number of meal components. This result is consistent with previous studies which showed a higher selectivity in chosen diet when a greater amount of forage is available [38]. It is important that coefficients of variation of the observed selection ratios are quite homogeneous for the preferred meal components (cereals and seeds) during the first hour throughout the tests, but relatively heterogeneous for the avoided silages. These were also lower during the first hour respect first to the fifth, suggesting that both selection in the second period and avoidance of silages during the whole experiment is quite variable. This variability probably depends on the availability and previous use of the preferred meal components.

Comparison between standardized selection ratios obtained during 1^st^ to 5^th^ hour respect to 1^st^ one (*i.e.*, *B_i_^1–5 h^* respect to *B_i_^0–1 h^*) increased for those meal components with increased availability, decreased for those with reduced availability and stayed stable when availability was similar (availability and *B_i_* values are shown in Tables 2 and 3). This is consistent with the diet-selection hypothesis which postulates that selectivity is a frequency-dependent process [50], [51]. However, selection ratio of meal components in both feeding periods correlated positively with energy and fat, and negatively with certain minerals (Ca, Na, Fe, B, Sr, and Ni). Thus, the existence of significant relationships among selection ratio and nutritive value of meal components is more consistent with another diet-selection hypothesis which suggests that ungulate diet selectivity is a complex process which involves both resource frequency and nutrient balancing [52]. This is further supported by the lower intensity in selecting high energy meal components when availability of resources decreases (lower slope in the correlation between energy content and standardized selection ratio *B_i_^1–5^* respect *B_i_^0–1^*; Figure 3).

In summary, resource availability, component frequency and energy content of components, all seem to influence selection index in our setting. Although both protein and energy are limiting nutrients for growth and reproduction [53], several studies have pointed out a higher selectivity for energy respect to protein and other nutrients, both in cervids [54]–[57] and other ruminants [58]–[60]. In fact, van Wieren [61] stated that 72% of diet selection by red deer could be explained by energy maximization. Rodriguez-Berrocal [62] also showed that the most selected plant species by free ranging Iberian red deer (*C. e. hispanicus*; heather and holm oak) were also the most energetic ones. Although protein percentage was relatively low in the offered diet for late pregnancy hinds (11.2% *vs.* 15% recommended by [63]; but see [64]), protein does not seem to be selected by ruminants or produce an intake increase as this would produce an interference with the metabolism of other nutrients [65]. Dinius and Baumgardt [66] showed that ruminants are able to adjust voluntary intake to meet energy demands when given pellet diets of varying energy content. Asher et al. [64] even found a great influence of energy in total daily intake irrespective of protein content when it is just above 8% in lactating red deer hinds. However, there is a general increase in energy intake for diets up to 12 000 kJ/kg [67], particularly if diet provided in our experiment has lower energy than recommended for late pregnancy hinds (2.1 *vs.* 2.9 kcal/kg recommended by [68]).

Finally, the positive selection found for neutral detergent fiber has been also previously documented [57]. Selection for starch has been scarcely studied, although it is the most important energetic component in diet of high-producing dairy cows (as source of glucose for ruminal microbial protein synthesis [68]. Meanwhile, minerals were negatively selected in our study. Several studies have shown the capacity of ruminants to select balanced diets of minerals (Belovsky [69] in moose *Alces alces*; Grassman and Hellgren [70] in *O. virginianus*). Red deer also seems to be able to discriminate them in diet [71] and consume them according to their individual requirements when offered as single mineral presentations without other nutrients [18]. However, ruminants respond more strongly to daily requirements for energy and protein [60], and selection for mineral use to be low when they are not deficient in diet [72]. Thus, in summary, our study conducted with the same deer herd and nutrition plane as Ceacero et al. [18], [71] indicate that deer can discriminate and consume minerals according to needs if offered alone, but they seem to select for energy when consuming complex food mixtures, at least in our setting with no serious mineral deficiency.

### Conclusion

In conclusion, deer compete for food even under *ad libitum* feeding conditions when food is offered under access restriction. In this situation, the aim by dominant hinds appears to be to ensure a longer early feeding time needed to select the preferred (and more energetic) food items. Although previous research has shown the ability to discriminate a range of essential minerals offered as salts, deer seem to select mainly for greater energy content and not minerals when facing a complex mixture of foods differing in energy, protein and mineral content, at least under the high nutrition plane of our setting. These results are important for feeding practices in captive deer herds, but also to understand feeding tactics and benefits of social rank in free-ranging herds when facing to seasonal starvation in the wild.
